# Supplementary material for: Patterns of Intron Gain and Loss in Fungi
Source: PLoS Biol. 2004 Nov 30;2(12):e422. doi: 10.1371/journal.pbio.0020422 (PMC532390; doi:10.1371/journal.pbio.0020422)
Supplement: Table S1 — Also available at http://genes.mit.edu/NielsenEtAl/. (4.3 MB ZIP). [file pbio.0020422.st001.zip › NielsenEtAl/html/984.html]

AN1433.1.NCU00761.1.MG10414.1.FG03601.1


```
 CLUSTAL W (1.82) Multiple Sequence Alignments - Introns Inserted


Sequence 1: AN1433.1	540 aa
Sequence 2: FG03601.1	537 aa
Sequence 3: NCU00761.1	608 aa
Sequence 4: MG10414.1	536 aa
Alignment Length: 625 aa
Number Identitical Residues: 153 aa
Alignment Score (without introns) 9700


MG10414.1 	------MARTLWKLALLGALPGLSSAALSTVVGDSCNQYAGTANATTGVTRWLGIRYASP
NCU00761.1	MRAVFAVAFQLFLSTIIISQLALTATCVRIVVNVTYSEYVG-EELGNDVSQWLGLRYAAP
FG03601.1 	--------MQSVSRLLLFAALALPSVAVDSTVDLGYSKYKG-KDLGNGVSEWLGVRYGAA
AN1433.1  	----------MLRPLAVLTALSALVSARDVVVDLGYARYRG-EALSNGVAQWLGIRYAAP
          	                : :  .    .   .*.    .* *     ..*:.***:**.:.

MG10414.1 	PVGSLRFMPPQDAPCQNGIQNATQ0HGPICIGTGE-SESATRSED--CLFLDIYAPSNAT
NCU00761.1	PVGALRFAPPQDPPSNSEPQAANK0HGNWCVRR---PGNSLTSED--CLFLDVYAPTQAT
FG03601.1 	PVKDLRWTLPQDPSRVRAVQDATK0RKPVCIGTSSDPSVIGNTEDEDCLFLNIWAPTHAS
AN1433.1  	PVGSLRFAAPQDPEDEYEIQDATQ0HGPVCIRASRYPIPEGSSED--CLFLDVYAPARPR
          	**  **:  ***.      * *.: :   *:  .  .     :**  ****:::**:.. 

MG10414.1 	SSSK--LPVVMFIQGGGFNKNSNSNYDGTGLVKAGDYGLIAITFNYRVGPYGFLTNGNEV
NCU00761.1	TKSN--LPVFVFIQGGGFNDNANPNLNGTGLVKASSNSIIVVTLNYRIGPYGFLTNGQQV
FG03601.1 	SKSK--LPVYFYIQGGGFNTNANPNVNGTALVKAGDLDMIVISIHYRVGVYGFLNDGADI
AN1433.1  	RDSSRLLPVYVFIQGGGFSSNGNPYYNGSGLIQASDMNIVVVNFNYRVGPYGFLAGSEIL
          	 .*.  *** .:******. *.*.  :*:.*::*.. .::.:.::**:* **** ..  :

MG10414.1 	EP---NNGLKDQRKALQWIKKNIAQ~FGGDPDHVVLIGGSAGGASISLQLIAYGGKDEGL
NCU00761.1	VA---NNGLRDQRKALEWVQKYISQ~FGGNPDHVVLGGASAGAASVAYHMMADKDGNRKL
FG03601.1 	KP---NLGLHDQRKALKWVQKYVAK~FGGNPDHVVIGGDSAGAASVSLHLSAYGGKDEGL
AN1433.1  	EGGSLNNGLRDQIQVLKWVQKHISK0FGGNPDHVVIGGDSAGGASVTLLLSAYEGRDDGL
          	  .: * **:** :.*:*::* ::: ***:*****: * ***.**::  : *  . :  *

MG10414.1 	FHGAAATAVSFATVLTVEESQYQYDNFAILAGCA---VADSLACLRSKNTTELQAANKPS
NCU00761.1	FHAAAAESVSFGTVLTVQQAQYQYDNLMIRLGCARSDAAASLTCLRSKTQQEIADKDGDI
FG03601.1 	FHGASAESISFGTLLTPKESSYQYQNLAIRLGCA-GPKKDVLRCIRSKSPQDIQKVNNAI
AN1433.1  	FHAAAAESQSFATMLTVDESQFAYDNLVNRTGCS-DTG-DTLNCLRSLDVEDLQRENIVT
          	**.*:* : **.*:** .::.: *:*:    **: .     * *:**    ::   :   

MG10414.1 	AYPGASEAPLYMWNPVVDGDMLTELTYTAFEEGRFLR-VPVIFGTSTNDGRSFAPRGTST
NCU00761.1	PYPGSSNGPIYMWSPVIDGDFVIDYPYSAFRNGNFIKNIPVIFGDDTNGGSGFVPDRISS
FG03601.1 	PFEGSSKAPLYMWTVSLDNDLIPDLTYKLFEQGKFIK-VPLITGDDTNGGTPFAPRDAAS
AN1433.1  	PFPGAQQPPLYMYGPTIDGDLIPDYTYRLFQQGKFIH-VPVIFGDVTNEGTKFVPEDLVS
          	.: *:.: *:**:   :*.*:: : .*  *.:*.*:: :*:* *  ** *  *.*    :

MG10414.1 	LGQSNTFLKDQFPYMTIEQMGKINDMYPNENN---TCPDTGCYFGQAAAAYGDVRYMCPG
NCU00761.1	LGDSNQFIKDQFPAITLSQLDTLNMLYPNPNAS--ICPKMGCWRGQAGSVYGEMRYMCPG
FG03601.1 	IGDSDEFLKAQFPFLTLEQLGEINELYPNKNN---TCPNAGCYWRQASTAYGDMRYMCPG
AN1433.1  	DTATYVWLQSQWPALTPKHFSRIHEYYLQDVNSTRPFPNATRYWRPVSDAYGEIRYVCPG
          	   :  ::: *:* :* .::. ::  * :   ::   *.   :   .. .**::**:***

MG10414.1 	MWISSIYEKFG----QGSKSWNYRYDVEDPKQVASGEGVPHT0SENQAIFGPE------Y
NCU00761.1	LYLNDAFDNYNDQYSKGTSSWAYRWNVEDRDQMASGLGVPHV~VELNALFGPANM----W
FG03601.1 	LYISNALTKYG-----VSKSWNYWYNVEDPAEMAQGLGVPHV~AELNAIFGPG------S
AN1433.1  	IALNAAYAKAG-----IPN-WNYHYAVQDPEDEASGLGVYHV~VELNAIWGPDNIWWSDT
          	: :.    : .      .. * * : *:*  : *.* ** *.  * :*::** .   :. 

MG10414.1 	TNGGAPASLYPGQINAHAVDVTQGYWTSFIRTLDPNTHKVADAVNWDTWT-----DAGHK
NCU00761.1	YTGQVPQSYFPGATNAAAVQVMQSYWVSFIRTFNPNTLRCCGGVEWKAWKSGDEASAQHQ
FG03601.1 	GNNNPPASYFPGEKNGPVIPVVQGYWSSFIRSLDPNTHRHEGSVKWETWTEKG-----KE
AN1433.1  	ISQPPPESYFT--FNAPIVPVMQGYWTSFIRSFDPNKHRYRGSPRWETWGKQ-------E
          	 .   * * :.   *.  : * *.** ****:::**. :  .. .*.:* .        :

MG10414.1 	RLLIATGGKSSMEVV----------~---------------------------------D
NCU00761.1	RLLFGTGGKTAMEVIPFDEGLGLRC1VAGSAWALNSRIIDGLAVPRFTQHSYGDCPSAVQ
FG03601.1 	RIVFETGGSTKMEKTS---------~----------------------------------
AN1433.1  	RLFVRTG-ETRMERVP---------~----------------------------------
          	*:.. ** .: **  .                                            

MG10414.1 	DKTRERCEYITSIG--VSIKQ--------
NCU00761.1	TERRPRPYRVPRAAGNVSISKNRKASSLT
FG03601.1 	QELQQKCKYLSSIG--ADIRQ--------
AN1433.1  	ADQRERCEYFISIG--IDLRQ--------
          	 . : :   .   .   .: :
```
